# Bacteriophages limitedly contribute to the antimicrobial resistome of microbial communities in wastewater treatment plants

**DOI:** 10.1128/spectrum.01101-23

**Published:** 2023-09-19

**Authors:** Raffaella Sabatino, Tomasa Sbaffi, Periyasamy Sivalingam, Gianluca Corno, Diego Fontaneto, Andrea Di Cesare

**Affiliations:** 1 Molecular Ecology Group (MEG), National Research Council of Italy – Water Research Institute (CNR-IRSA), Verbania, Italy; Chung-Ang University, Anseong, South Korea

**Keywords:** antimicrobial resistance, bacteriophages, metagenomics, metagenomic assembled genomes

## Abstract

Bacteriophages are known as players in the transmission of antimicrobial resistance genes (ARGs) by horizontal gene transfer. In this study, we characterized the bacteriophage community and the associated ARGs to estimate the potential for phages to spread ARGs in aquatic ecosystems analyzing the intra- and extracellular DNA isolated from two wastewater treatment plants (WWTPs) by shotgun metagenomics. We compared the phage antimicrobial resistome with the bacterial resistome and investigated the effect of the final disinfection treatment on the phage community and its resistome. Phage community was mainly composed by *Siphoviridae* and other members of the order *Caudovirales*. The final disinfection only marginally affected the composition of the phage community, and it was not possible to measure its effect on the antimicrobial resistome. Indeed, only three phage metagenome-assembled genomes (pMAGs) annotated as *Siphoviridae*, *Padoviridae*, and *Myoviridae* were positive for putative ARGs. Among the detected ARGs, i.e., *dfr*B6, *rpo*B mutants, and EF-Tu mutants, the first one was not annotated in the bacterial MAGs. Overall, these results demonstrate that bacteriophages limitedly contribute to the whole antimicrobial resistome. However, in order to obtain a comprehensive understanding of the antimicrobial resistome within a microbial community, the role of bacteriophages needs to be investigated.

**IMPORTANCE:**

WWTPs are considered hotspots for the spread of ARGs by horizontal gene transfer. In this study, we evaluated the phage composition and the associated antimicrobial resistome by shotgun metagenomics of samples collected before and after the final disinfection treatment. Only a few bacteriophages carried ARGs. However, since one of the detected genes was not found in the bacterial metagenome-assembled genomes, it is necessary to investigate the phage community in order to gain a comprehensive overview of the antimicrobial resistome. This investigation could help assess the potential threats to human health.

## INTRODUCTION

Antimicrobial-resistant bacteria (ARB) and antimicrobial resistance genes (ARGs) are widely distributed in aquatic environments (1, 2), and some of them are by now recognized as constitutively present in autochthonous aquatic bacterial communities (3). However, other ARGs can be found within aquatic microbial communities as a direct consequence of human activities, and they can be reduced or absent if the anthropogenic impact is decreased (4). Particularly relevant, in this respect, is the role of the wastewater treatment plants (WWTPs), which mirror the antimicrobial resistance gradient observed in clinics (5) and are considered among the main hotspots for the release of ARB and ARGs in open water and sediments (6, 7). WWTPs are currently not designed to lower the ARG abundances, and the impact of the applied treatments on them is only indirect. Furthermore, WWTPs, stressing the microbial communities and promoting ecological and environmental instability in a short time frame, are also considered hotspots for horizontal gene transfer (HGT) (8).

HGT implies that bacteria can horizontally receive ARGs by conjugation, transformation or transduction. The last two HGT mechanisms do not require cell-cell contact, and in particular, the transduction is mediated by bacteriophages. Bacteriophages (phages) are “the smallest and simplest biological entities” (9), and, at the same time, they are abundant in very different environments: 10^15^ viral particles in the “phageome” (total content of the human gut phage particles) (10), >10^7^ viral particles per mL in coastal seawater (11). Moreover, they are among the drivers of the evolution of bacterial genomes by HGT (12). HGT could, thus, involve ARGs, and, although its frequency in the environment is unknown (13), it poses bacteriophages as potential players for the spread of ARGs into the environment with a nowadays unknown influence on the cycle of ARB in the frame of the *One Health* concept.

In this study, we investigated the composition of the phage community and its ARG content in treated wastewaters pre- and post-final disinfection in order to understand: (i) if and to which extent phages are carriers of ARGs, (ii) if the ARGs carried by phages are different than those hosted by the bacterial community, and (iii) if the final disinfection treatment could affect the bacteriophage composition and the associated antimicrobial resistome.

We selected two WWTPs in Northern Italy, one located in Verbania (VB) and another one in Cannobio (CB). They are characterized by different wastewater inflows, population equivalent (PE), and disinfection treatment. The WWTP of VB treats domestic and pre-treated hospital sewages, serves 51,000 PE, and applies chlorination as final disinfection, while the WWTP of CB treats domestic and industrial sewages, serves 15,000 PE, and uses peracetic acid for the final disinfection. Wastewaters were collected before and after the final disinfections and processed for the extraction of intra- and extracellular DNA (i-and eDNA). The DNA samples were previously sequenced by shotgun metagenomics (14), and the phage metagenome-assembled genomes (pMAGs) were retrieved and annotated at the taxonomic level, using a recently developed pipeline that recovers viruses from bulk metagenomic data without enrichment, classifying non-phage bins with very high accuracy (93%–99%) (15). Thus, the antimicrobial resistome was determined.

## RESULTS

### Phage community composition

**Fig 1:**
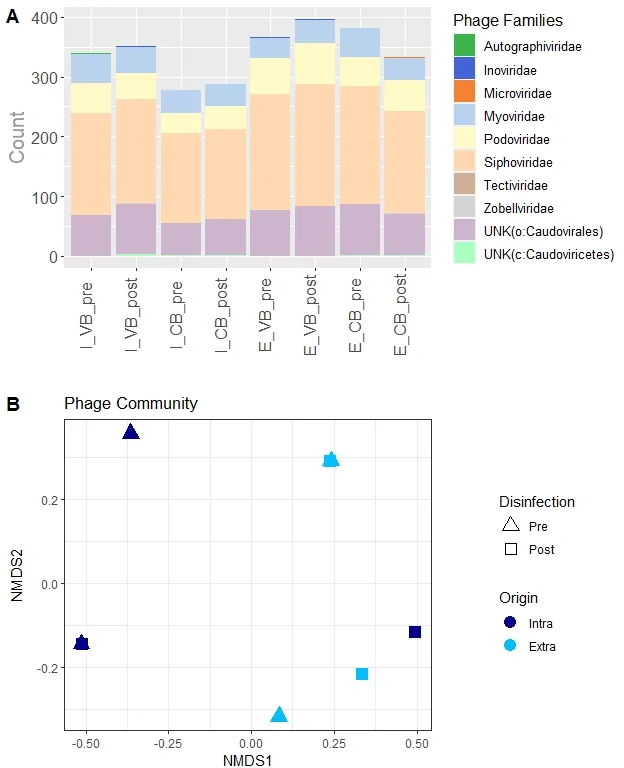
Phage community composition. Community composition of the samples according to (**A)** phage families of the retrieved MAGs, and (**B)** NMDS analysis. In cases with no possible assignation to the family level, the family is labeled as “unknown” (UNK), and the closest assigned taxonomic level is reported between brackets.

A total of 2,734 pMAGs were retrieved (Table S1), covering eight different viral families (Fig. 1A). 2,145 pMAGs were assigned to family level, 582 to order level, and 7 only to class level (Fig. 1A). The majority (80%) of recovered pMAGs represented dsDNA phages and, to a lesser extent (20%), ssDNA viruses. We recovered 1,255 pMAGs in the iDNA samples and 1,479 in the eDNA ones (Table S1). Richness did not differ significantly among the samples neither when considering the WWTP and the disinfection as factors (nested ANOVA: *P* ≥ 0.444) nor the origin of phages: iDNA or eDNA (*t* test: *P* = 0.486). 48.2% of differences in the taxonomic composition of phages (beta diversity) were explained in a PERMANOVA by the origin of phages (iDNA or eDNA), whereas the WWTP (25.3%) and, even less, the disinfection step (9.8%) had a minor role in shaping the community composition (Fig. 1B). No correlation was observed between the composition of phage communities from intra- and extracellular samples (Mantel test: *r* = −0.2, *P* = 0.71).

### ARGs in phage and bacterial MAGs

**Fig 2:**
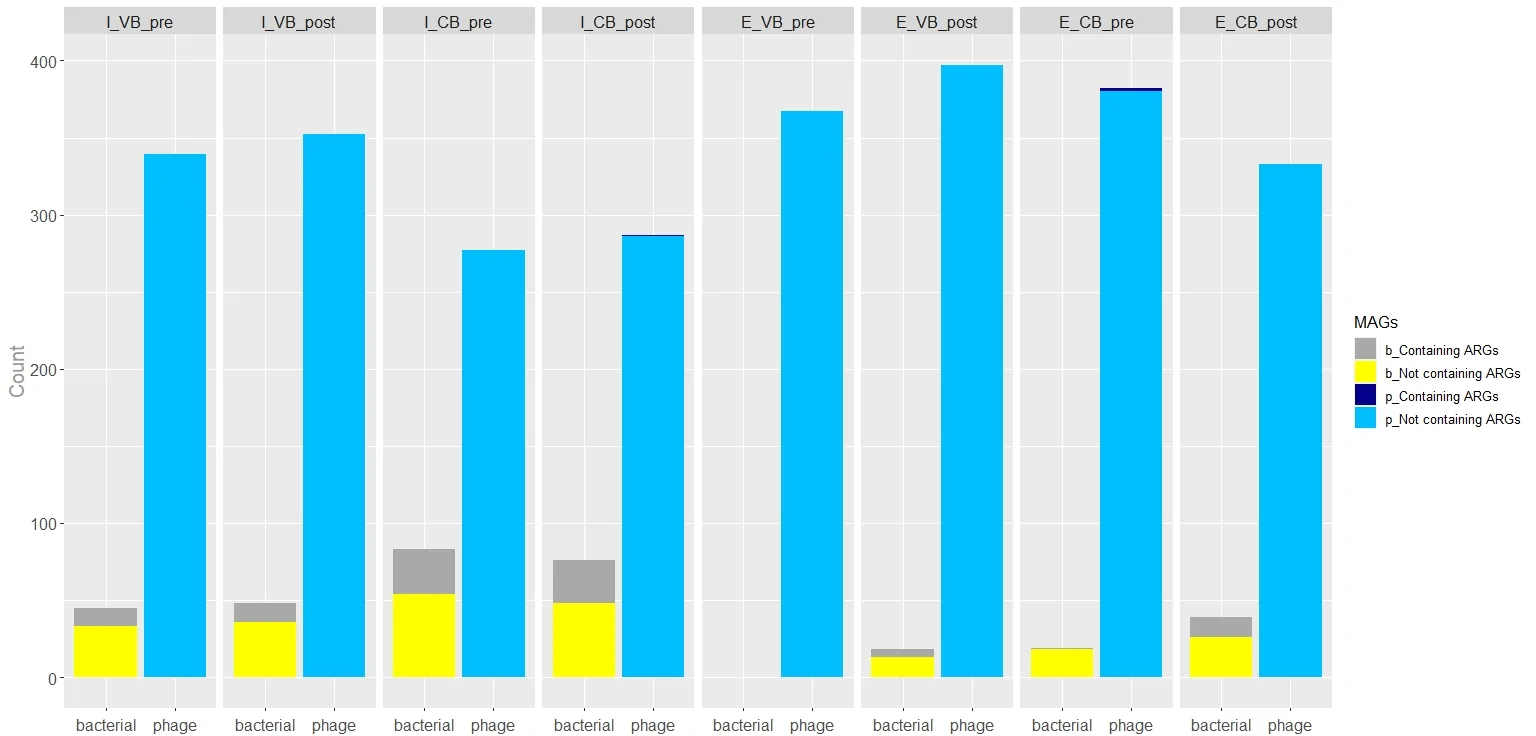
Antimicrobial resistome in phage and bacterial MAGs. The count of the retrieved bacterial and phage MAGs, comprising the number of the ones positive for at least one ARG, is depicted. In the legend, the first lowercase letter represents bacterial (“b”) or phage (“p”) MAGs. In the sample name, the first letter (“I” or “E”) indicates the origin of phage DNA (intra- or extracellular), “VB” or “CB” codes identify the WWTP (of Verbania or Cannobio, respectively), and “pre” and “post” labels refer to samples at pre- and post-disinfection step, respectively.

**Fig 3:**
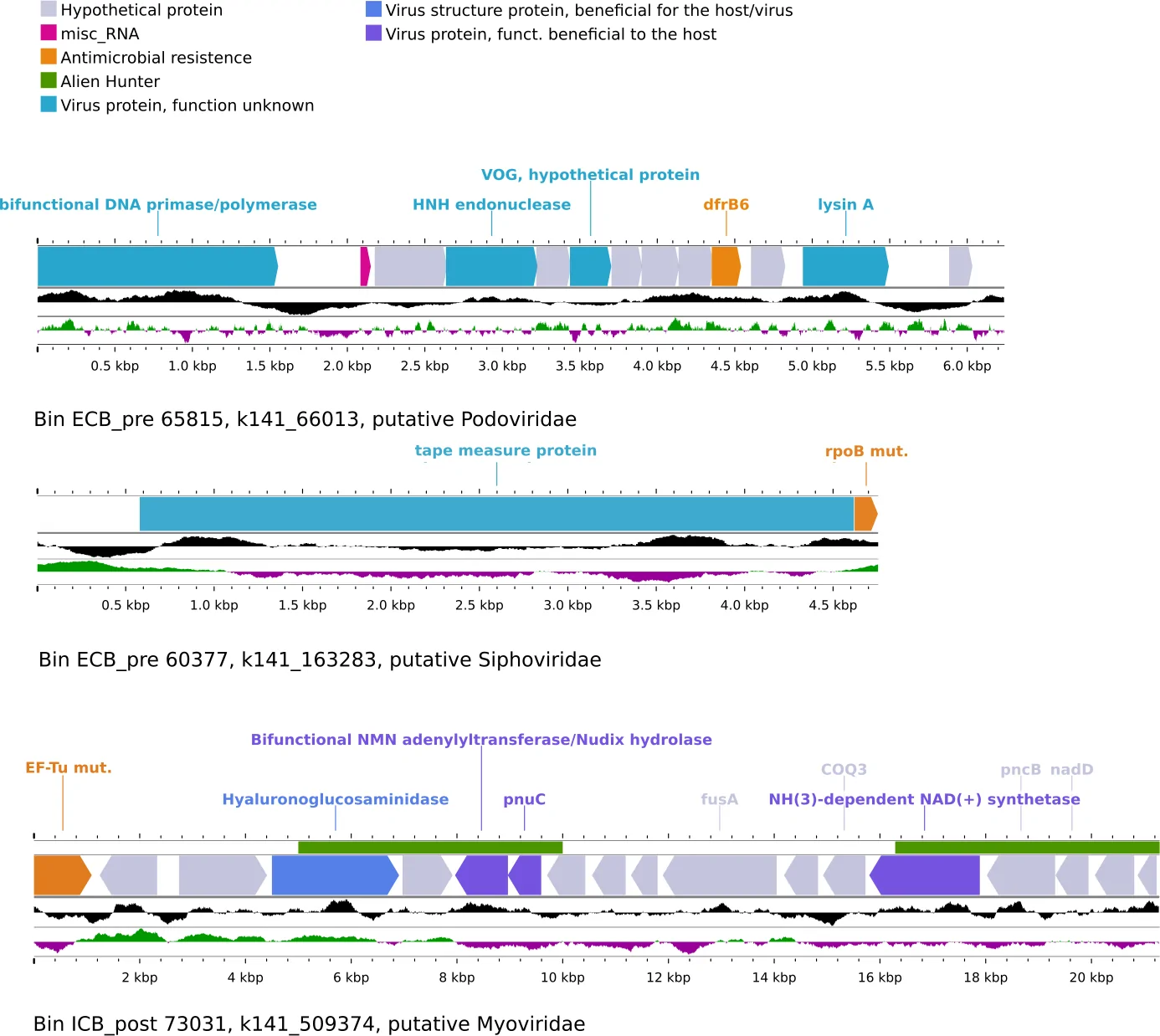
Linear representation of ARG-containing phage contigs. Focus on the contigs showing the presence of ARGs in three bins. The genomic map was obtained with Proksee (https://proksee.ca). The upper track indicates horizontal gene transfer (HGT) putative regions predicted by Alien Hunter, forward and reverse ORFs are represented in the same tracks, the underlying track shows the skewed content in GC along the sequence, and putative viral genes were obtained by annotating with the VOG database as part of the PHAMB pipeline.

Out of 2,734 pMAGs, three were positive for at least one ARG (Fig. 2, details of the ARG-containing phage contigs are shown in Fig. 3). The *Myoviridae*, *Siphoviridae*, and *Podoviridae* families were found as carriers of ARGs (Table 1). One ARG-positive pMAG was detected in the intracellular fraction of DNA, and two were observed in the extracellular one (Table 1). All the pMAGs were recovered from WWTP located in CB and none from the WWTP located in VB (Table 1). Two of the pMAGs carrying ARGs were observed in pre-disinfection samples and only one in post-disinfection samples (Table 1). Among the ARGs detected in pMAGs, we found *Bifidobacterium adolescentis rpo*B mutants (hereafter, *rpo*B mut), encoding resistance against rifampicin of the rifamycin antibiotic class, *dfr*B6, encoding resistance against the diaminopyrimidine antibiotic class, and *Escherichia coli* EF-Tu mutants, encoding resistance against Pulvomycin (EF-Tu mut) of the elfamycin antibiotic class (Table 1). The antibiotic resistance function of *dfr*B6 and *rpo*B mutants cannot be definitively determined; thus, they are here referred as putative ARGs. In particular, the sequence here annotated of the *rpo*B mut gene does not include the rifampicin resistance-determining regions (16). However, it shows several aminoacidic mutations if compared with the wild-type *rpo*B protein sequence of *Mycobacterium tuberculosis* (GeneBank accession number L27989) (17) (Table S2). No metal resistance genes (MRGs) and mobile genetic elements (MGEs) were annotated in the ARG-positive phage contigs. In a previous work analyzing the same water samples, we reconstructed 328 bacterial metagenome-assembled genomes (bMAGs) (14), of which 100 were positive for at least one ARG (Fig. 2): 81 bMAGs in the iDNA and 19 in the eDNA (Table S1). Twenty-nine of the bMAGs carrying ARGs were found in the samples from the WWTP of VB and 71 in the samples from the WWTP of CB (Table S1). Forty-two of the ARG-positive bMAGs were present in pre-disinfection samples and 58 in post-disinfection samples (Table S1). The elfamycin resistance class was the most frequently detected resistance (found in 85 bMAGs and represented by EF-Tu mut in 75 bMAG), followed by the resistance genes encoding resistance to more than one antibiotic class (in nine bMAGs), and fluoroquinolone and rifamycin resistance (in seven and six bMAGs, respectively; Table S1). Other detected resistance classes accounted for a smaller extent (found in less than five bMAGs; Table S1).

**TABLE 1:** Phage MAGs positive for ARGs^*a*^

Sample	Bin	Order	Family	Contig (ORF)	Bitscore	Identity	Gene	Sequence variation	Resistance mechanism	Antibiotic class
ECB_pre	60,377	Caudovirales	Siphoviridae	k141_16328_3	75.485	76.74	*B. adolescentis rpo*B mutants conferring resistance to rifampicin	n/a	Antibiotic target alteration; antibiotic target replacement	Rifamycin
ECB_pre	65,815	Caudovirales	Podoviridae	k141_66013_9	92.0485	75.44	*dfr*B6	n/a	Antibiotic target replacement	Diaminopyrimidine
ICB_post	73,031	Caudovirales	Myoviridae	k141_509374_1	530.02	70.72	*E. coli* EF-Tu mutants conferring resistance to Pulvomycin	R234F	Antibiotic target alteration	Elfamycin

^*a*^ In the sample name, the first letter (“I” or “E”) indicates the Origin of phage DNA (intra- or extracellular), “CB” codes identify the WWTP of Cannobio, and “pre” and “post” labels refer to samples at pre- and post-disinfection step, respectively. n/a = not available.

## DISCUSSION

The main factor influencing the beta diversity of the phage community was the origin of DNA, i.e., intra- or extracellular. Furthermore, the phage community composition detected in the intracellular DNA was not correlated with that of the extracellular one. These findings highlight that both fractions of DNA are needed to completely characterize the phages within and outside the bacterial cells. The final disinfection treatment of the WWTPs only limitedly affected the composition of the phage community, similarly to what we observed for the bacterial community (14), suggesting that chemical disinfection has only a marginal role in shaping it. The phage community was composed mainly by dsDNA viruses belonging to the order *Caudovirales* in agreement to previous results from WWTPs processed by shotgun metagenomics (18, 19). This result is not surprising and could be reasonably due to the wide distribution of these tailed bacteriophages in human feces (20). *Siphoviridae* was the most abundant family: members of this family infect a wide range of bacteria, including those that are potentially pathogenic for humans, e.g., *E. coli*, *Streptococcus*, and *Staphylococcus* (21). Despite our data set being limited to a small number of samples, comprising approximately 2,700 pMAGs, we found that *Siphoviridae*, *Podoviridae* and *Myoviridae* were the only viral families that tested positive for three ARGs. This finding, similar to what was previously obtained in hospital wastewater samples (22), suggests that these viral families may have the potential to facilitate the lateral transfer of ARGs. Only a very limited fraction of pMAGs was positive for ARGs, as also observed in other environments, e.g., human and mouse feces (23), pig feces (24), and soils (25). The number of ARGs annotated in the pMAGs was more than 30-fold lower than that found in bMAGs of the same wastewater samples (14), confirming the trend observed in other WWTP samples (26, 27).

However, it is important to note that one putative ARG, i.e., *dfr*B6, annotated in a pMAG, was not detected in the bMAGs. This result emphasizes the significance of characterizing bacteriophage communities to obtain a comprehensive understanding of the antimicrobial resistome present within a DNA sample. *dfr*B6 was previously detected in viruses from environmental samples (19, 25). Furthermore, this gene is particularly interesting, considering that it was previously found on MGEs in pathogenic bacteria (28). The other detected putative ARG, *rpo*B mut resulted previously correlated with bacteriophages in sewage samples (29). The last detected ARG, EF-Tu mut, has been previously extensively found in eDNA in wastewater samples (30). However, among our samples, only a pMAG retrieved from iDNA was positive for this mutated gene. Furthermore, to the best of our knowledge, it has not been previously annotated in bacteriophages. Therefore, this is the first report that this gene resulted to be carried by bacteriophages.

Overall, these findings indicate that phages within wastewater microbial communities may play a role in spreading ARGs. While their contribution to the whole antimicrobial resistome may not be relevant in quantitative terms, they play a significant role by harboring a distinctive ARG that is not detected when analyzing bMAGs. Thus, phages contribute to the diversity and spread of ARGs, highlighting their importance in the broader context of antimicrobial resistance in wastewaters.

## MATERIALS AND METHODS

### Study design

In November and December of 2021, 3 h integrated wastewaters pre- and post-disinfection from two WWTPs located in Piedmont (Northern Italy) were collected and processed for the intra- and extracellular DNA extraction. The DNA samples were sent to IGA Technology Services Srl (Udine, Italy) for shotgun metagenomic sequencing, and the raw sequence data were submitted at NCBI under accession number PRJNA881852. The sampling, DNA extraction, and sequencing were previously done and published (14).

### Bioinformatic analyses

#### Processing of metagenomic co-assembled data and virus binning from bulk metagenome

Assembled data derived from Sivalingam et al. (14) were used for the present study. Concisely, after quality filtering performed with TrimGalore! v0.6.5 (https://github.com/FelixKrueger/TrimGalore), co-assembly was performed using MEGAHIT v1.2.9 (31), with the “meta-sensitive” preset and a minimum contig length of 1,000 bp. Co-assemblies were defined by date, as per reference 14 considering that no statistically significant differences were found for this variable and the resulting co-assemblies were IVB_pre, IVB_post, ICB_pre, ICB_post, EVB_pre, EVB_post, ECB_pre, and ECB_ post, where I and E indicate the intra- or extracellular DNA origin, VB or CB highlights the location of the sample (VB or CB) and -pre or -post indicate if the sample is pre- or post-disinfection. The processed reads were mapped to the co-assembled metagenomic contigs using BWA-MEM algorithm v 0.7.17 with default parameters (32).

The virome was studied through the newly developed PHAMB pipeline (15), based on contig binning to retrieve viral genome bins from bulk metagenomics data, allowing the clustering of putative pMAGs discerning those from bMAGs. The PHAMB pipeline relies on a classifier that can classify non-phage bins from any data set with very high accuracy (93%–99%) (15). We performed the pipeline using the eight co-assembled metagenomes, singularly. Briefly, the function jgi_summarize_bam_contig_depths from Metabat2 v2.15 (33) was used to calculate contigs abundances for each co-assembly, while the deep-learning algorithm for metagenomic binning VAMB v3.0.2 (34) was specifically used for the binning process. The PHAMB workflow relies on a set of steps aimed at searching bins content for hallmark viral and bacterial genes/proteins. Viral predictions on contigs of minimum 1,000 bp were run using DeepVirfinder (v1.0, default settings) (35), VOGdb (https://vogdb.org/), and MicompleteDB (36) via hmmsearch v3.3.2 function (37), using the flag -E 1.0e-5, on predicted ORFs (open reading frames) by prodigal v2.6.3 (38) (-p meta -g 11). The outputs of the mentioned annotations served as input to the PHAMB random forest model (run_RF.py) that was run on binned clusters. The quality of generated pMAGs was assessed using the average amino acid identity model of CheckV v1.0.1 (39), and only high quality or complete viral and proviral pMAGs (completeness >90%) were retained for further analyses (15). The non-viral regions in pMAGs were removed by CheckV.

#### Phage taxonomy

Viral proteins were predicted with prodigal using the –meta preset. Consequently, proteins were annotated using the viral protein-specific databases VOGdb or the viral subsets of TrEMBL used in the tool Demovir (https://github.com/feargalr/Demovir). Phage taxonomy was assigned to each bin using the plurality rule described before in reference 40 as suggested and performed in reference 15. For both methods, we followed the guidelines provided by the authors.

#### Phage genes and ARGs annotation of putative pMAGs and bMAGs

Previously predicted proteins from putative pMAGs and bMAGs were aligned to the CARD database v.3.2.7 using the rgi tool v 6.0.2 (41), which uses BLASTp (42) [--include_loose –include_nudge, identity 70% (43, 44), and bitscore ≥50 (45)]. Best matches to the CARD database were reported as counts for each co-assembly. The phage contigs displaying the presence of ARGs were subsequently scanned for the co-presence of MRGs by alignment with the BACmet database v 2.0.15 with DIAMOND blastp (--more-sensitive --id 70 -e 1e-10) and the MEGARes databases v 3.0.0, with blastn (42) (-evalue 1e-10 -perc_identity 70 -word_size 5). To further investigate the presence of genes indicating the presence of integrons and MGEs, the selected phage contigs were aligned to INTEGRALL (46) and mobileOG (47) databases using, respectively, blastn (-evalue 1e-10 -perc_identity 70 -word_size 5) and the related tool mobileOGs-pl-kyanite.sh (-e 1e-10 -p 80 -q 60). The predicted ORFs annotated as viral-like genes against the VOGdb as part of the PHAMB pipeline were highlighted in the genomic map. All genomic maps were built with Proksee (https://proksee.ca). The tool Alien Hunter available in Proksee was used to highlight putative HGT regions of the contigs identified as “alien” and therefore considered to originate from HGT (48).

The whole bioinformatic workflow described in this work is available at https://github.com/TomasaSbaffi/Bulk_metagenome_virome_2023.

### Data analyses

The statistical analyses were made in the R environment v 4.1.2 (49), considering eight samples. For the tests, we used as explanatory variables the WWTP (two levels, VB and CB), the step of the disinfection process (two levels, “Pre” and “Post” disinfection), and the origin of phages (two levels, “Intra” and “Extra” cellular DNA) and performed nested analyses to take into account the hierarchical nature of the sampling activities, with two types of origin of phages nested within two types of disinfection steps, nested within the two WWTPs. Thus, after having retrieved the pMAGs, we determined the richness (as a number of different MAGs) of each sample and evaluated which factors (WWTP/Disinfection/Origin) influenced its variation through a nested ANOVA for the WWTP/Disinfection and then applying a *t*-test for the Origin. Similarly, differences in beta diversity, as abundance-based Bray-Curtis dissimilarity index, were investigated using a nested PERMANOVA, first, and a PERMANOVA with strata within paired (Intra-Extra) samples, successively. Non-metric Multidimensional Scaling (NMDS) analyses were carried out and used to plot sample composition. Moreover, the beta diversity dissimilarity matrix was subset into two sub-matrices (containing only “Intra” and “Extra” samples, respectively), and these ones were correlated by Mantel test, using Pearson’s correlation method, to unveil potential concordance or discordance in the community composition of extra- and intracellular phages.

## Data Availability

The raw sequence data were submitted at NCBI and are publicly available under accession number PRJNA881852. Furthermore, the wild type *rpoB* protein sequence of *Mycobacterium tuberculosis* is publicly available under the GenBank accession number L27989.

## SUPPLEMENTAL MATERIAL

Supplemental tables spectrum.01101-23-s0001.xlsx Tables S1 and S2.

ASM does not own the copyrights to Supplemental Material that may be linked to, or accessed through, an article. The authors have granted ASM a non-exclusive, world-wide license to publish the Supplemental Material files. Please contact the corresponding author directly for reuse.
